# A pilot study of aquatic prehabilitation in adults with knee osteoarthritis undergoing total knee arthroplasty – short term outcome

**DOI:** 10.1186/s12891-021-04253-1

**Published:** 2021-04-26

**Authors:** Sunghye Kim, Fang-Chi Hsu, Leanne Groban, Jeff Williamson, Stephen Messier

**Affiliations:** 1 Department of Internal Medicine, Section of Rheumatology, Wake Forest School of Medicine, Winston-Salem, NC USA; 2 Department of Medicine, Section of Rheumatology, W.G. Hefner VA Medical Center, Salisbury, NC USA; 3 Division of Public Health Sciences, Department of Biostatistics and Data Science, Wake Forest School of Medicine, Winston-Salem, NC USA; 4 Department of Anesthesiology, Wake Forest School of Medicine, Winston-Salem, NC USA; 5 Sticht Center on Aging, Wake Forest School of Medicine, Winston-Salem, NC USA; 6 J.B. Snow Biomechanics Laboratory, Department of Health and Exercise Science, Wake Forest University, Section on Gerontology and Geriatric Medicine, Wake Forest School of Medicine, Winston-Salem, NC USA

## Abstract

**Background:**

Knee osteoarthritis (KOA) is increasingly more prevalent and significant number of patients require knee arthroplasty. Although knee arthroplasty is generally successful, it takes months to recover physical function. Preoperative physical function is known to predict postoperative outcomes and exercise can improve preoperative physical function. However, patients with KOA have difficulty exercise on land due to pain and stiffness, while water exercise can be better tolerated. We hypothesized that preoperative water exercise to improve preoperative physical function will improve postoperative outcomes after total knee arthroplasty (TKA).

**Methods:**

We enrolled 43 participants who were scheduled for elective TKA in 4–8 weeks and scored at or below 50th percentile in mobility assessment tool-sf (MAT-sf). All enrolled participants were assessed on 1) clinical osteoarthritis symptom severity using Western Ontario and McMaster Universities Osteoarthritis Index (WOMAC), 2) physical function using Short Physical Performance Battery (SPPB), 3) self-reported mobility using Mobility Assessment Tool-short form (MAT-sf), 4) depression using Geriatric Depression Scale-short form (GDS-sf), 5) cognitive function using Montreal Cognitive Assessment (MoCA). Blood samples for high-sensitivity-C-reactive protein (hs-CRP), tumor necrosis factor-alpha (TNF-α) and interleukin-6 (IL-6) were stored at − 80 °C then all samples were analyzed together. All the enrolled participants were randomly assigned to the aquatic exercise intervention (AEI) or usual care group. Sixty minute sessions of AEI was conducted three times a week for 4–8 weeks. Participants in both groups were evaluated within 1 week before their scheduled surgery, as well as 4 weeks after the surgery.

**Results:**

The mean age was 67.1 (±6.2), 44% were female, 74% were White. There is no statistically significant difference in combined outcome of any complication, unscheduled ER visit, and disposition to nursing home or rehab facility by AEI. However, AEI was associated with more favorable outcomes: WOMAC scores (*p* < 0.01), chair-stand (*p* = 0.019), MAT-sf as well as improved depression (*p* = 0.043) and cognition (*p* = 0.008).

**Conclusion:**

4–8 weeks of aquatic exercise intervention resulted in improved functional outcomes as well as improved depression and cognition in elderly patients undergoing TKA. A larger study is warranted to explore the role of water exercise in clinical and functional outcomes of TKA.

## Background

With an aging population, knee osteoarthritis (KOA) is increasingly more prevalent; it affects 37% of older adults aged 60 years and older [1]. A significant number of patients with advanced KOA require knee arthroplasty and this procedure is expected to increase by 143% from 2012 to 2050 [2]. Although knee arthroplasty is generally successful in limiting pain and improving function, it takes at least 6 months to recover physical function [3] and a significant number of patients experience persistent pain and disability after the procedure [4]. Preoperative physical function is known to predict postoperative outcomes [5].

Pain with weight bearing is a universal symptom of KOA and with progression of KOA, pain limits mobility and functionality. This immobility from pain can contribute to weight gain, which in turn enhances further progression of KOA. This cycle is especially true in patients with advanced KOA, such as patients who are considered for a total knee arthroplasty (TKA).

Immobility and weight gain caused by pain can affect postoperative outcomes after TKA in several ways. First, mobility limitation is known to be a risk factor for worse postoperative outcomes; our prospective cohort study discovered that preoperative mobility limitation, assessed by the Mobility Assessment Tool-short form (MAT-sf), is associated with longer postoperative lengths of stay, higher postoperative complication rates, and greater nursing home placement after major surgery. Second, elderly patients with limited mobility quickly develop declines in physical function [6] that are well known risk factors for postoperative complications and poor postoperative function [7].

*Prehabilitation* is defined as enhancing the functional capacity of an individual to enable him/her to withstand an incoming stressor [8]. Multiple studies have tested the effect of prehabilitation in patients scheduled for total joint replacement surgery. A systematic review of 35 studies with 2956 patients reported that prehabilitation was associated with improved postoperative function after total hip arthroplasty (THA) or TKA as well as significantly shorter length of stay after TKA [9]. While land exercise is easier and more convenient to perform, it is challenging for patients with knee osteoarthritis since weight bearing can cause pain in patients with advanced KOA who already have significant pain and stiffness. Water exercise allows less impact to joints due to buoyancy and it can be an alternative to land-based exercise in patients with advanced KOA who are undergoing TKA. There is some evidence that aquatic exercise leads to a short term improvement in pain and disability in patients with hip or knee osteoarthritis [10].

We designed this pilot clinical trial to test our hypothesis that improving preoperative physical function using aquatic exercise intervention (AEI) in elderly patients with advanced knee osteoarthritis (KOA) who are undergoing TKA will improve postoperative outcomes.

## Methods

### Study design

This was a pilot randomized clinical trial.

### Inclusion and exclusion criteria

We recruited participants aged 50 years and older who are scheduled for primary TKA. Although the initial inclusion criteria was participants aged 65 years and older, due to poor enrollment, we changed the inclusion criteria in April 2016. We screened participants in the Preoperative Assessment Clinic at Wake Forest Baptist Medical Center. A trained study coordinator screened potential participants for baseline mobility status, using the mobility assessment tool-short form (MAT-sf). Those participants who score at or below the 50th percentile, based on our prior preoperative study data, were considered eligible (e.g. MAT scores ≤58 for men and ≤ 50 for women). Exclusion criteria included patients who were undergoing knee replacement for indications other than osteoarthritis, revision surgery or bilateral surgery, scheduled for emergency surgery; afraid of water or are not willing to undergo water exercise; having major deficits in hearing or vision; currently exercising more than 3 times a week; participating in another clinical trial; or cannot understand the questionnaires and directions due to cognitive impairment or language barriers. Following these tests, participants underwent a standard medical workup at the discretion of the PAC’s attending physician, including American Society of Anesthesiologists (ASA) physical status scoring, and Revised Cardiac Risk Index (RCRI) classification. For each participant, information regarding demographic characteristics, comorbidities, medications were gathered.

### Assessment

Blood pressure, pulse, height and weight were assessed. All enrolled participants were assessed by a trained study coordinator on 1) Western Ontario and McMaster Universities Osteoarthritis Index (WOMAC) pain, stiffness, and physical function [11], 2) physical function using the Short Physical Performance Battery (SPPB), 3) self-reported mobility using Mobility Assessment Tool-short form (MAT-sf) 4) depression using Geriatric Depression Scale Short Form (GDS-sf), and 5) cognitive function using Montreal Cognitive Assessment (MoCA). The WOMAC (score 0–96) is a widely used and validated tool to assess pain, stiffness, and physical function in patients with hip and/or knee osteoarthritis. It has three subscales including pain (5 items, score 0–20), stiffness (2 items, score 0–8) and physical function (17 items, score 0–68) [11]. The SPPB consists of repeated chair stands, balance testing, and 4-m walking speed (total score 0–12). It is known to predict subsequent disability, institutionalization, and mortality [12]. The GDS-sf (score 0–15) is a 15 item screening tool for depression for older adults and it has been validated in the preoperative setting. Score of 0–5 is considered as normal, while a score of > 5 suggests depression [13]. The MAT-sf is a 10-item computer based assessment of mobility using animated video clips [14]. The 10 items in the MAT-sf cover a broad range of functioning. The items include walking on level ground, a slow jog, walking outdoors on uneven terrain, walking up a ramp with and without using a handrail, stepping over hurdles, ascending and descending stairs with and without the use of a handrail, and climbing stairs while carrying bags. It has been validated against measures of physical function, including the Pepper Assessment Tool for Disability, the Short Physical Performance Battery, and 400-m walk test among a population of older community dwellers. The Montreal Cognitive Assessment (MoCA) is a widely validated 30 points test that measures several domains of cognitive function, including short-term memory recall tasks, visuospatial abilities, executive function, attention, concentration, working memory, language, and orientation [15]. Baseline blood were drawn at the first visit for high-sensitivity-C-reactive protein (hs-CRP), tumor necrosis factor-alpha (TNF-α) and interleukin-6 (IL-6) and, stored at − 80 °C until the completion of the study and all samples were analyzed together.

Data to calculate Charlson comorbidity [16] was obtained by chart review and the score was calculated.

### Intervention arms

After baseline assessments, all the enrolled participants were randomly assigned to the intervention or control group, using a pre-generated randomization list. The AEI were conducted in warm water (a minimum of 90 °F) at the Wake Forest Baptist Medical Center Therapy Pool, three times a week, and for 60 min each session until the scheduled surgery (4–8 weeks). The duration of the intervention was determined based on the duration between the preoperative visit with orthopedic surgeon and the surgery date. To maximize the enrollment, we designed the study that would not interfere usual practice pattern. All the sessions were supervised by an aquatic therapist. The AEI was designed to improve 1) range of motion, 2) muscle strength, and 3) resistance, resulting in improvement in mobility in this low-mobility population. The protocol consists of a warm-up (10 min); joint range of motion for flexibility and strength (20 min); low intensity endurance such as walking to prevent chilling and maintain and/or improve cardiovascular fitness (20 min); and end with a cool down (10 min). Depending on the participant’s tolerance, resistance equipment, such as a noodle and/or neoprene ankle cuff were added to increase the exercise intensity. The participants’ exercise log was kept by the aquatic exercise director to record the compliance to the protocol. A trained study coordinator called the intervention group participants weekly to assist with problem solving, reinforce progress, and to encourage adherence. Control group participants received a brochure on perioperative nutrition in addition to the standard cares, such as optimization of their underlying medical problems and perioperative infection prevention measures.

### Reevaluation before surgery

Participants in both groups were evaluated 1 week prior to their scheduled surgery. Blood pressure, pulse, and weight were assessed. Symptoms of osteoarthritis, self-reported mobility, depression, and cognition using the WOMAC, MAT-sf, GDS-sf and MoCA were assessed by a study coordinator. The SPPB was administered by two designated trained study coordinator who were not supervising the aquatic exercise and were masked to the randomization status. At this time, follow up labs were drawn to measure hs-CRP, TNF-α, and IL-6.

### Reevaluation after surgery

Participants in both groups were asked to return 4–6 weeks after the scheduled surgery. The follow up time was chosen so the visit can coincide the patient’s visit with their postoperative visit with their surgeon. Blood pressure, pulse, and weight were assessed. Osteoarthritis symptoms, self-reported mobility, depression and cognition, using WOMAC, MAT-sf, GDS-sf and MoCA were assessed by a study coordinator. Physical function was assessed using SPPB by two designated trained study coordinators who were not supervising the aquatic exercise and were masked to the randomization status. Follow up labs were drawn to measure hs-CRP, TNF-α, and IL-6. The follow up duration was decided based on the primary outcome of interest − 30 day National Surgical Quality Improvement Project (NSQIP) defined morbidity and mortality as well as hospital length of stay, ICU length of stay, delirium, and institutionalization.

### Outcome assessment

The primary outcome of interest was 30 day National Surgical Quality Improvement Project (NSQIP) defined morbidity, including surgical site infection, wound disruption, pneumonia, unplanned intubation, pulmonary embolism, ventilator > 48 h, acute renal failure, urinary tract infection, stroke, coma, peripheral nerve injury, cardiac arrest, myocardial infarction, requirement of transfusion, deep vein thrombosis, sepsis and mortality [17], hospital length of stay, ICU length of stay, delirium, and institutionalization. The secondary outcomes were osteoarthritis symptoms using WOMAC, self- reported mobility measured by MAT-sf, physical function, measured by SPPB, depression, measured by GDS, cognition, measured by MoCA, and inflammatory profiles, including hs-CRP, TNF-α, and IL-6.

### Power calculation

The power calculation for detecting the intervention effect on the unfavorable outcomes was done using the two proportion test. Assuming 20 participants in each group (in this study, the sample size is 20 in the intervention group and 23 in the standard care group), a two-sided test and a significance level of 0.05, we have 80% power to detect an odds ratio of 6.6 and 9 when the proportions of unfavorable outcome are 10 and 30% in the intervention group, respectively. We have limited power in this analysis. If we could increase the sample size to 100 per group in future studies, then the detectable odds ratios become 3.0 and 2.3, respectively. Additionally, we note that enrolling 20 per group provides 80% power to detect mean differences (via analysis of covariance) of 1.04, 0.88, 0.68, 0.39 standard deviation in outcomes between the two intervention groups, assuming a correlation coefficient between the outcome measure at baseline and at follow-up equals to 0.3, 0.5, 0.7, and 0.9, respectively, given a two-sided test and a significance level of 0.05. We have more power to detect the differences when the correlation coefficient is larger than 0.5. If we could increase sample size to 100 per group, the detectable differences are 0.47, 0.39, 0.31, 0.18 standard deviation in outcomes between the two intervention groups, assuming a correlation coefficient of 0.3, 0.5, 0.7, and 0.9, respectively. In general, the effect sizes are reasonably small when sample size is 100 per group for future trials.

### Statistical analysis

All statistical analyses were performed using SAS software, version 9.4 (SAS Institute, Cary, NC). Sample means and standard deviations were computed for the continuous baseline characteristics, and counts and proportions were calculated for the discrete baseline characteristics according to intervention groups. For non-normally distributed characteristics, medians, 1st quartile, and 3rd quartile were also calculated. Logistic regressions were used to examine the associations between intervention groups and unfavorable outcomes, including postoperative complications, longer hospital stay of over 2 days, disposition to nursing home/rehab after adjusting for anesthesia time. Odds ratios (ORs) and their 95% confidence intervals (CIs) were presented. In order to best approximate the conditional normality assumption, the distributions of functional measurements were checked and transformed if needed. Differences in mean values of each functional measurement between intervention groups were estimated using repeated measures analysis of covariance with baseline outcome measure, intervention assignment, visit, and an intervention by visit interaction included in the model. Hypothesis tests for intervention effects at the follow-up visits were performed using contrasts. Overall comparisons between groups across follow-up visits were obtained using a contrast to compare average intervention effects across both follow-up visits. Raw mean and standard deviation were presented at baseline, adjusted least squares means and standard error were presented at post-intervention and post-operative visits.

## Results

**Fig. 1:**
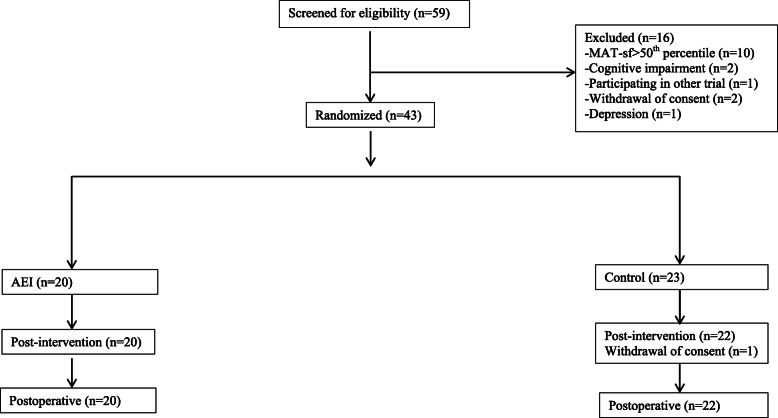
Screening and follow up

We screened 59 patients and randomized 43 participants. Figure 1 demonstrates the flow diagram. Table 1 demonstrates the baseline characteristics of the study subjects. There was no difference by the group at baseline.

**Table 1:** Baseline characteristics by intervention groups

	Intervention group (*n* = 20)	Standard care group (*n* = 23)	Overall (*n* = 43)
Age (years)	67.4 ± 6.0	66.9 ± 6.3	67.2 ± 6.1
Female (%)	10 (50%)	9 (39%)	19 (44%)
White (%)	17 (85%)	16 (70%)	33 (74%)
Black	2 (10%)	7 (30%)	9 (21%)
BMI, mean (kg/m^2^)	32.9 ± 7.4	31.9 ± 5.3	32.4 ± 6.3
Charlson’s comorbidity score	3.74 ± 0.73	3.80 ± 1.28	3.77 ± 1.04
hs-CRP	5.72 ± 7.48	2.37 ± 3.57	3.93 ± 5.90
	2.81 (0.63–7.59)^a^	1.17 (0.73–2.46)^a^	1.97 (0.69–3.33)^a^
TNF-α	1.38 ± 0.58	2.85 ± 6.43	2.17 ± 4.73
	1.27 (0.94–1.55)^a^	1.26 (0.81–1.56)^a^	1.26 (0.85–1.56)^a^
IL-6	4.16 ± 4.04	2.68 ± 2.46	3.37 ± 3.34
	2.84 (1.59–5.09)^a^	2.16 (1.40–2.77)^a^	2.28 (1.47–3.94)^a^

^a^median (Q1-Q3)

The median number of water exercise sessions was 12 (IQR = 12 to 16) over a 44 (median 29) day period. The median anesthesia time was 144.5 min (IQR =129 to 158). After total knee arthroplasty, 13.2% of patients experienced any complications including superficial, deep and organ space surgical site infection, wound disruption, pneumonia, unplanned intubation, pulmonary embolism, ventilator > 48 h, progressive renal insufficiency, acute renal failure, urinary tract infection, stroke, cardiac arrest, myocardial infarction, deep vein thrombosis, return to the operating room, systemic sepsis. The median length of stay was 2 days (IQR = 2 from 0 to 2) and 2 (5.3%) patients were discharged to nursing home or rehabilitation facilities. Logistic regression analyses were performed for composite outcome of various unfavorable outcomes, including any complications, unscheduled ER visit, disposition to nursing home/rehab facility, length of stay > 2 days, or combined outcomes. Water exercise was not associated with any of the outcomes (Table 2).

**Table 2:** Multivariable logistic regression analysis of unfavorable outcomes and intervention groups after controlling for anesthesia time

	OR	95% CI	*P* value
Any complication	1.5	0.21–11.2	0.68
Unscheduled ER visit	0.13	0.01–1.3	0.08
Disposition to nursing home/rehab	0.43	0.04–4.24	0.47
LOS > 2 days	0.17	0.02–1.83	0.14
Combined outcomes^a^	0.23	0.04–1.17	0.08

^a^Combined outcomes = any complication, unscheduled ER visit, disposition to nursing home or home with health care (vs. home without health care)

Table 3 demonstrates the summary statistics of outcome measurements at baseline, post-intervention and post-operative. Water exercise was associated lower systolic and diastolic blood pressure at post-operative visit although it did not result in statistically significant weight loss. Water exercise was associated with improved WOMAC score (− 11 points) and all three subscales of WOMAC- pain, stiffness and physical function with more significant difference at post-intervention visit although the association was attenuated at postoperative visit compared to post-intervention visit.

**Table 3:** Association between the functional measurements and intervention group over time using repeated measures analysis of covariance^a^

	Visits	Mean (95% CI)^b^		*P* value for intervention by visit interaction	*P* value for intervention effect at each follow-up visit	Overall intervention effect (95% CI), *p*-value
		Control	AEI			
Weight (kg)	Baseline	92.5 ± 15.5	93.8 ± 21.8	0.77		−4.9 (−11.9, 2.1), 0.16
	Post-intervention	94.3 ± 2.4	89.3 ± 2.5		0.16	
	Post-operative	92.3 ± 2.5	87.0 ± 2.6		0.16	
SBP	Baseline	137.9 ± 13.6	131.0 ± 20.5	**0.020**		−6.3 (−15.0, 2.4), 0.15
	Post-intervention	133.8 ± 3.5	134.1 ± 3.6		0.94	
	Post-operative	141.3 ± 3.6	127.9 ± 3.6		**0.014**	
DBP	Baseline	75.3 ± 10.4	72.0 ± 10.4	**0.026**		−2.7 (−6.6, 1.2), 0.17
	Post-intervention	72.9 ± 1.7	73.9 ± 1.8		0.69	
	Post-operative	77.6 ± 1.8	70.9 ± 1.8		**0.014**	
PP	Baseline	68.7 ± 11.0	65.3 ± 7.7	0.59		−3.6 (−7.4, 0.2), 0.061
	Post-intervention	72.7 ± 1.5	69.7 ± 1.6		0.18	
	Post-operative	78.3 ± 2.4	73.1 ± 2.4		0.14	
WOMAC-total	Baseline	44.9 ± 10.7	49.4 ± 14.9	0.90		−11.1 (−15.7, −6.5), **< 0.001**
	Post-intervention	48.9 ± 1.7	37.7 ± 1.8		**< 0.001**	
	Post-operative	39.2 ± 3.2	28.6 ± 3.3		**< 0.026**	
WOMAC-pain	Baseline	8.6 ± 3.0	10.0 ± 3.7	0.86		−2.0 (−3.2, −0.8), **0.002**
	Post-intervention	9.3 ± 0.4	7.3 ± 0.5		**0.0029**	
	Post-operative	8.2 ± 0.7	6.4 ± 0.7		0.0654	
WOMAC-Stiffness	Baseline	4.3 ± 1.3	4.3 ± 1.2	0.17		−0.87 (−1.36, − 0.38), **0.001**
	Post-intervention	4.5 ± 0.2	3.3 ± 0.3		**0.0013**	
	Post-operative	4.0 ± 0.2	3.5 ± 0.2		0.1417	
WOMAC- function	Baseline	32.0 ± 8.2	35.0 ± 11.0	0.88		−7.6 (−11.6, −3.6), **< 0.001**
	Post-intervention	35.0 ± 1.5	27.6 ± 1.6		**0.0003**	
	Post-operative	26.8 ± 2.7	18.7 ± 2.7		0.8764	
SPPB-total	Baseline	8.4 ± 2.1	8.4 ± 2.3	0.75		0.94 (−0.31, 2.20), 0.14
	Post-intervention	8.7 ± 0.5	9.5 ± 0.5		0.18	
	Post-operative	7.4 ± 0.5	8.5 ± 0.6		0.17	
SPPB- balance	Baseline	3.6 ± 0.8	3.6 ± 0.9	0.35		0.04 (−0.49, 0.56), 0.88
	Post-intervention	3.6 ± 0.2	3.6 ± 0.2		0.88	
	Post-operative	3.6 ± 0.2	3.8 ± 0.2		0.55	
SPPB- gait speed	Baseline	3.1 ± 1.0	3.0 ± 1.0	0.96		0.22 (−0.25, 0.69), 0.36
	Post-intervention	3.1 ± 0.2	3.4 ± 0.2		0.37	
	Post-operative	2.6 ± 0.2	2.8 ± 0.2		0.49	
SPPB- chair-stand	Baseline	1.7 ± 1.2	1.9 ± 1.4	0.99		0.70 (0.21, 1.19), **0.006**
	Post-intervention	1.9 ± 0.2	2.6 ± 0.2		**0.019**	
	Post-operative	1.2 ± 0.3	1.9 ± 0.3		0.094	
MAT-sf	Baseline	48.0 ± 6.6	45.7 ± 5.8	0.79		4.34 (0.90, 7.79), **0.015**
	Post-intervention	44.8 ± 1.2	49.0 ± 1.3		**0.019**	
	Post-operative	45.1 ± 2.0	50.1 ± 2.0		0.090	
GDS-sf	Baseline	1.7 ± 1.3	1.9 ± 2.1	0.35		−1.02 (−2.08, 0.04), 0.058
	Post-intervention	2.9 ± 0.4	1.7 ± 0.4		**0.043**	
	Post-operative	2.8 ± 0.5	2.3 ± 0.5		0.51	
MoCA	Baseline	26.0 ± 1.8	25.5 ± 2.2	0.73		1.46 (0.46, 2.45), **0.005**
	Post-intervention	25.7 ± 0.4	27.2 ± 0.4		**0.008**	
	Post-operative	26.2 ± 0.4	27.6 ± 0.4		**0.033**	
Log hs-CRP	Baseline	0.32 ± 0.96	0.87 ± 1.46	0.34		−0.25 (−0.69, 0.20), 0.27
	Post-intervention	0.79 ± 0.15	0.58 ± 0.15	0.35		
	Post-operative	1.80 ± 0.28	1.23 ± 0.29	0.17		
Log TNF-α	Baseline	0.33 ± 0.89	0.25 ± 0.38	0.88		0.10 (−0.07, 0.26) 0.25
	Post-intervention	0.19 ± 0.07	0.27 ± 0.07		0.39	
	Post-operative	0.26 ± 0.07	0.36 ± 0.07		0.32	
Log IL-6	Baseline	0.77 ± 0.61	1.08 ± 0.84	0.051		−0.02 (−0.32, 0.28), 0.90
	Post-intervention	0.94 ± 0.11	1.04 ± 0.11		0.56	
	Post-operative	1.53 ± 0.12	1.31 ± 0.13		0.23	

^a^Baseline outcome measure, intervention assignment, visit, and an intervention by visit interaction were included in the model

^b^raw mean and standard deviation for baseline, adjusted least squares means and standard error for post-intervention and post-operative visits

There was no association between water exercise and physical function measured by total SPPB. However, water exercise was associated with improved with chair- stand, one of the subscales of SPPB, especially at post-intervention visit. Water exercise was also associated with improved self-reported mobility, measured by MAT-sf. GDS-sf sores increased in both groups at the post-operative visit (more depression symptoms), although still in normal range. Subjects in the AEI group had less increase in GDS-sf score compared to the control group. Water exercise was also associated with improved cognition, measured by MoCA.

There was no association between AEI and any of the inflammatory markers- (hs-CRP, IL-6, and TNF-α).

To explore the effect of inflammation in mediating the association between water exercise and functional outcomes, we fit two models with and without each inflammatory markers. Other than the effect of CRP’s effect on the water exercise and walk speed, there was no mediating effect of any of the inflammatory markers and functional outcomes.

## Discussion

In this pilot clinical trial of 43 participants who are undergoing total knee arthroplasty for advanced osteoarthritis, 10–24 sessions of preoperative water exercise over 4–8 weeks was associated with lower blood pressure, better osteoarthritis symptoms measured by WOMAC although we had limited power. The association was more prominent at the post-intervention preoperative period, and the association was generally attenuated after surgery except blood pressure which seems to be only prominent in post-operative period.

Water exercise was also associated with better mobility, mood (lower depression score) and cognition. However, water exercise did not result in better postoperative outcomes.

Our study found that water exercise resulted in improved osteoarthritis symptoms with decrease in total and each individual WOMAC subcategories of pain, stiffness and function. The total WOMAC score decreased by 11 which is higher than the minimum clinically important difference after total knee arthroplasty [18]. In a Cochrane review of 54 randomized controlled trials of land-based exercise for knee osteoarthritis, exercise has shown to reduce pain, improve physical function and quality of life immediately after treatment [19] and the 2019 American College of Rheumatology osteoarthritis guidelines strongly recommended exercise as a treatment for osteoarthritis [20]. Water exercise might be a better choice for patients with osteoarthritis. Buoyancy provided by water offers less impacts on joints while land work a significant barrier in exercise among patients with OA [19] while land base exercise can cause significant pain in patients with osteoarthritis. In a study of 126 participants with knee OA and at least one morbidity found that a 20-week individualized, comorbidity adapted land-based exercise program resulted in improvement in WOMAC physical function score of 7.43 [21] while our study of 4–8 weeks of water exercise intervention saw a similar effect (WOMAC physical function score improvement of 7.6) with much shorter intervention time. Another study of 8 weeks of aquatic physiotherapy in 60 participants with knee osteoarthritis reported improvement in total WOMAC score by 14.2 [22]. This improved WOMAC score continued even in the post-operative period with a difference of 11, suggesting that AEI not only improves preoperative but post-operative outcomes of pain, stiffness and function. A study of 87 post-menopausal women with mild knee osteoarthritis reported that 4 months of water resistance training resulted in decrease in WOMAC stiffness domain of 8.5 compared to control group although the benefit does not maintain after the intervention is over [23]. Although these water-based exercise studies have reported improved outcomes, in clinical setting, access to facilities with therapy pool as well as the willingness of patients, especially older patients’ to participate in water exercise might be an issue.

AEI was also associated with chair-stand in SPPB and self-reported mobility, although the association was attenuated after scheduled TKA. It is hypothesized that our study subjects have advanced KOA and the improved symptom and function by our water exercise was improvement is not as potent as knee replacement surgery. Future studies of water exercise in less advanced KOA might demonstrate the effect of intervention at earlier stages of KOA can result in delay in the need for arthroplasty. Our hypothesis was that water exercise would improve preoperative physical function that is known to be a predictor of postoperative outcomes. In the elderly population, preoperative frailty, physical function, preoperative exercise capacity, and mobility are reported to be predictive of surgical outcomes [7, 24–28]. Prehabilitation using exercise is thought to improve postoperative recovery and physiologic reserve by increasing aerobic capacity and muscle strength [29, 30]. The lack of association between water exercise prior to total knee arthroplasty and favorable postoperative outcomes could be explained by our relative small sample size (*n* = 43). Future larger studies might find an association between preoperative water exercise and improved postoperative outcome. Another possibility is that our intervention in our study might have not been long enough. Prior studies used exercise of 1 day to 8 weeks [9]. The AEI group subjects participated in 12 water exercise sessions (IQR = 12 to 16) over a 44 (median 29) day period. Although the dose–response relationship between preoperative exercise and postoperative outcomes is not established, a longer duration of intervention might result in more beneficial postoperative outcomes. Another possibility is that our intervention group was at higher risk for poor postoperative outcomes. Although there was no statistical significance, the baseline hs-CRP was numerically higher in the intervention group. Elevated CRP was reported as a predictor of postoperative surgical site infection [17], postoperative infection [31], and general complication rate [32]. Given our relatively small sample size, we could not include too many co-variates in the model.

Hypertension is a very common condition, especially in the elderly- 63% of the population aged 60 and over [33]. On average, our study subjects had a baseline blood pressure in the pre-hypertensive range. Interestingly, the systolic blood pressure in the intervention group at the post-operative visit decreased by 3.1, while in the control group, it increased by 2.4. The blood pressure lowering effect of AEI was significant 4 weeks after surgery, while there was no difference in the post-intervention (preoperative) period. Exercise has been known to be effective in lowering blood pressure [34] . This was reported in a clinical trial of water exercise study as well– the blood pressure lowering effect persisted 12 weeks after a 12-week training intervention [35]. The authors of the report hypothesized that the prolonged effect could be a consequence of immersion in heated water. It is not clear why the control group subjects in our study experienced an increase in their blood pressure while the intervention group subjects experienced a decrease in blood pressure. Also, given the wide variation of blood pressure at baseline (standard variation 13.6–20.5), the differences in blood pressure measures between groups that we observed in the study might not be significant and subclinical. However, if the trend of blood pressure continues, it can certainly contribute to long-term outcomes.

Water exercise was associated with a better GDS score. While the GDS-sf score increased in the post-intervention and post-operative period in both groups compared to the baseline, the increase in the water exercise group was not as significant. The association between exercise and improved depression was reported before [36], and it is hypothesized that exercise improves depression by increasing neurogenesis and triggering plastic processes [37]. However, it is also well known that pain and depression are closely related especially in elderly and common pathologic mechanisms including neuroinflammation is hypothesized to play a role in both conditions [38]. Since there is evidence that physical exercise improves neuroinflammation it is possible that our intervention resulted in lower levels of neuroinflammation and subsequent improvement in depression [39]. Although the increase in GDS-sf in post-intervention and post-operative period is subclinical, there is a known association between geriatric depression and worse outcomes in many medical conditions and its association with disability [40], so further study in occurrence of perioperative depression and possible interventions is warranted.

The subjects enrolled in our study had a baseline MOCA score that was close to the mild cognitive impairment cut off of 26 [15] and water exercise group subjects had improved MOCA scores after intervention. A systematic review with multilevel meta-analysis of 36 studies reported the association between physical exercise and cognitive function [41]. However, there are other studies that reported no or only very mild effect of physical exercise on cognitive function [42, 43]. Our study demonstrated that the MoCA score of the intervention group improved by 1.7 and 2.1 in the post-intervention and post-operative period, while the score did not change in the control group. In a study of 51 healthy middle aged women, 45 min water exercise sessions twice a week for 6 months period was associated with improved cognition [44]. It was hypothesized that exercise intervention, especially aerobic exercise improves cardiorespiratory fitness which in turn improves brain perfusion as well as decreases systemic inflammation and oxidative stress [45]. Another possibility is the association between pain and cognition– patients with pain can present with cognitive complaints [46]. Water exercise resulted in improved pain and that might explain the improvement in cognition measured by MoCA.

There was no association between inflammatory markers, including hs-CRP, TNF-α, and IL-6 levels, and AEI. The baseline hs-CPR was numerically higher in the AEI group, but it was not statistically significant and there was no association between AEI and any of the markers.

### Limitation

Our study has a few limitations. First, the sample size is relatively small (*n* = 43) to detect the treatment effect of water exercise. This limited power might be a reason that we did not find any difference in postoperative outcomes in the intervention group vs control group. This small sample size also did not allow us to create multi-variate models with multiple co-variates. Also, we only followed the patients 4–6 weeks after the surgery. It is known that the functional recovery after TKA occurs over months to a year [47, 48], and it is possible that after the 4 weeks follow up the participants’ functional outcomes (physical, cognitive, mood) might have different trajectories. We designed current pilot study to evaluate if aquatic exercise intervention in elderly patients with advanced knee osteoarthritis (KOA) who are undergoing TKA can improve postoperative short term outcomes including postoperative complications, unscheduled ER visit, disposition to nursing home/rehabilitation facility and length of stay. Although current study can’t predict long term functional outcomes of after TKA, it is known that fast pain response (fast pain relief) at 8 weeks after TKA could predict improved pain at 6 month, suggesting short term postoperative outcome can predict long term outcome [49]. However, there is another study that reported three types of postoperative pain and function recovery patterns- “high risers”, “gradual progressors” and “non-responders” [50], making long term prediction with short term outcome difficult. Future longer term follow up study is warranted to provide answers on the effect of preoperative aquatic exercise intervention in long term functional outcomes after TKA. We enrolled participants who are willing to participate in aquatic exercise intervention. Accordingly, the participants in this study might not represent the general population who are undergoing total knee arthroplasty. Our study enrolled participants with poor mobility and the preoperative WOMAC score was higher than other study of patients who were undergoing total knee arthroplasty [51]. It is possible that out finding might not be applicable to patients who are undergoing TKA but do not have severe mobility limitation. Finally, the majority of our patients were white and it might not be generalizable to the general population.

## Conclusion

In this small pilot study of 43 subjects with advanced knee osteoarthritis undergoing total knee replacement surgery, 4–8 weeks of water exercise weeks was associated with lower blood pressure, better osteoarthritis symptoms measured by WOMAC, although it did not result in improvement in postoperative outcomes. The intervention was also associated with better self-reported mobility, physical function, mood as well as cognition, although the effect tends to be attenuated in the postoperative period. Water exercise was associated with lower systolic and diastolic blood pressure in the postoperative period although there was no effect of the intervention on weight. Future study of a larger population and less advanced osteoarthritis is warranted to explore the effects of water exercise on osteoarthritis symptoms as well as cognition, depression and blood pressure.

## Data Availability

The datasets used during the current study are available from the corresponding author on reasonable request.

## Abbreviations

KOA: Knee osteoarthritis
TKA: Total knee arthroplasty
MAT-sf: Mobility assessment tool-sf
WOMAC: Western Ontario and McMaster Universities Osteoarthritis Index
SPPB: Short Physical Performance Battery
GDS-sf: Geriatric Depression Scale-short form
MoCA: Montreal Cognitive Assessment
hs-CRP: High sensitivity C-reactive protein
TNF-α: Tumor necrosis factor-alpha
IL-6: Interleukin-6
AEI: Aquatic exercise intervention
ASA: American Society of Anesthesiologists
RCRI: Revised Cardiac Risk Index classification
NSQIP: National Surgical Quality Improvement Project
ICU: Intensive care unit
ORs: Odds ratios
CIs: Confidence intervals
IQR: Interquartile range
